# Exploring Unconventional Electron Distribution Patterns: Contrasts Between FeSe and FeSe/STO Using an Ab Initio Approach

**DOI:** 10.3390/ma17215204

**Published:** 2024-10-25

**Authors:** Chi-Ho Wong, Rolf Lortz

**Affiliations:** 1 Department of Physics, The Hong Kong University of Science and Technology, Hong Kong, China; 2 Division of Science, Engineering, and Health Studies, School of Professional Education and Executive Development (SPEED), The Hong Kong Polytechnic University, Hong Kong, China; 3 Department of Industrial and Systems Engineering, The Hong Kong Polytechnic University, Hong Kong, China

**Keywords:** ab initio, antiferromagnetic phonon, spin-density wave, charge-density wave

## Abstract

For more than a decade, the unusual distribution of electrons observed in ARPES (angle-resolved photoemission spectroscopy) data within the energy range of ~30 meV to ~300 meV below the Fermi level, known as the ARPES energy range, has remained a puzzle in the field of iron-based superconductivity. As the electron–phonon coupling of FeSe/SrTiO_3_ is very strong, our investigation is centered on exploring the synergistic interplay between spin-density waves (SDW) and charge-density waves (CDW) with differential phonons at the interface between antiferromagnetic maxima and minima under wave interference. Our analysis reveals that the synergistic energy is proportional to the ARPES energy range, as seen in the comparison between FeSe and FeSe/SrTiO_3_. This finding may suggest that the instantaneous interplay between these intricate phenomena may play a role in triggering the observed energy range in ARPES.

## 1. Introduction

Iron-based superconductors have attracted considerable attention in the field of condensed matter physics due to their unique properties and potential applications in high-temperature superconductivity [1,2,3]. However, there are numerous complex features that still lack a comprehensive understanding, such as the intricate effects of antiferromagnetism, nematicity, gap anisotropy, spin–orbit coupling, and other factors on the underlying pairing mechanism [1,2,3]. In particular, the experimental ARPES studies have confirmed the unusual electron distribution pattern below the Fermi level in most iron-based superconductors. When the electron distribution across the Fermi level follows a step function, it represents a typical scenario where the probability of finding an electron below the Fermi level is 1. Even at temperatures above 0 K, the distribution follows a hyperbolic tangent, though the energy range influenced by thermal excitation is narrow. In contrast, an unconventional electron distribution can be marked by the disappearance of electrons below the Fermi level [4,5], occurring over a much broader energy range, where the electrons within ~30 meV to ~300 meV below the Fermi level can be surprisingly affected by iron-based superconductivity [4,5,6,7,8]. In spite of extensive research efforts over the past decade, the origin of this unconventional electron distribution pattern has remained elusive even with AI analysis [9,10,11]. One challenge in applying AI to this problem is that an effective classification depends on identifying the contrasts between training data and output [9,10,11]. If researchers can pre-establish a correlation or feature between the ARPES energy range and the input data for iron-based superconductivity, it will be easier for AI to distinguish the weighed parameters for this problem more effectively [9,10,11].

In view of this, we explored the correlation between the ARPES energy range and the interplay of various electronic correlations, lattice structure, and key phenomena such as antiferromagnetism (AFM), spin-density waves (SDWs), and charge-density waves (CDWs) in the tetrahedral regions. The existence of spin-density waves can lead to a twofold enhancement $R_{SDW}=2$ in the local electron–phonon scattering matrix at AFM maxima [12]. This enhancement arises from either the conservation of AFM energy or the transfer of AFM energy from AFM minima to AFM maxima. As electrons traverse the regions with spin-density waves, they encounter a time-varying magnetic energy, according to Maxwell’s equation, which in turn generates an electric xy potential across the interface between the antiferromagnetic (AFM) and non-magnetic (NM) sites. This phenomenon can be described as a magnetoelectric pulse, and the resulting induced electric potential may be viewed as a charge-density-wave phenomenon. This CDW effect further contributes to an additional approximately twofold enhancement $R_{CDW}\;\sim \;2$ in the local electron–phonon scattering matrix [12]. In the presence of spin-density-wave (SDW) and charge-density-wave (CDW) effects, there is typically a differential out-of-plane phonon behavior of Fe atoms between the antiferromagnetic (AFM) maxima and minima (the AFM minima~non-magnetic (NM) sites). This means that the phonon speed under the AFM/NM sites is usually slower/faster, leading to an electron–differential phonon interaction as a consequence of antiferromagnetism, spin-density wave, and charge-density wave. This consideration results in a significant improvement in the agreement between simulation results and experimental observations [12,13].

Based on their preliminary work [12,13], this paper focuses on examining the synergistic effects of spin-density waves (SDWs) and charge-density waves (CDWs) with differential phonons, specifically at the interface between antiferromagnetic (AFM) and non-magnetic (NM) regions, and then investigate if the synergic energies have a correlation with the ARPES energy range. This study diverges from conventional approaches that primarily consider interactions with average phonons. The comparison of the synergistic interactions between FeSe and FeSe/SrTiO_3_ may create a contrast that helps to establish a correlation with the ARPES energy range [4,5,6]. This could enable AI to analyze the weighted factors contributing to the ARPES energy range among various parameters more easily.

## 2. Computational Methods

For a lattice ion located at position $R_{i}$, with a displacement $u_{i}(x,y,z)$ from its equilibrium position $R_{i}^{0}$, and assume that the periodic synergetic potential V(r) is rigid, the electron–phonon interaction $H_{e-ph}$ can be computed by the Jellium model [14]. In this work, the interaction $H_{synergy}$ between electrons and the differential phonon under the coexistence of antiferromagnetism, charge-density wave, and spin-density wave are considered.

Antiferromagnetism can enhance the electron–phonon interaction $H_{e-ph}$ by a factor of R_AF_^2^ [15,16,17]. In the presence of spin-density waves, the antiferromagnetic fluctuations are expected to form AFM local minima and maxima alternatingly [12] in the form of constructive-like interference. It is important to note that the square of the electron–phonon scattering matrix is proportional to the electron–phonon interaction [15]. The spin-density wave and charge-density wave associated with the appearance of a differential phonon modifies the interaction to be $H_{synergy}\;\sim \;H_{e-ph}\cdot R_{SDW}^{2}\cdot R_{CDW}^{2}\cdot R_{AF}^{2}$. The non-cancellable or differential vibrational amplitude along the out-of-plane axis (i.e., $\delta u_{i}(z)=|u_{i}(z_{peak})|-|u_{i+1}(z_{peak})|\neq 0$) of the nearest Fe neighbors occurs and eventually amends the electronic density of the states in the *xy* plane across the AFM–NM interface. The influence of exchange coupling to $H_{synergy}$ can be rewritten through the separation of variables, in which the exchange factor $f(E_{ex})\;\sim \;\frac{M_{Fe}^{2}E_{co}{|}_{P>0}}{M_{Fe}^{2}E_{co}{|}_{P=0}}$ is used to monitor the AFM fluctuations under pressure *P*, where $R_{AF}^{2}{|}_{P>0}\;\sim \;R_{AF}^{2}{|}_{P=0}\cdot f(E_{ex})$ and $R_{tetra}^{2}{|}_{P>0}\sim R_{tetra}^{2}|{|}_{P=0}\cdot f(E_{ex})$ [18].

The $H_{synergy}$ on the Fermi level is estimated for the compressed bulk FeSe and 2D FeSe/SrTiO_3_. We applied CASTEP (version 2022a) to conduct the spin-unrestricted calculations at the GGA-PW91 level [16,17]. The maximum SCF cycle was set to 100 with a tolerance of 2 µeV/atom, and the reciprocal k-space grid was 0.025 (1/Å). The ultrasoft pseudopotential was used. The phonon data was calculated by the finite displacement mode, where the cutoff radius is 0.5 nm, and the interval of the dispersion is 0.04 (1/Å). The non-cancellable or differential vibrational amplitude along the out-of-plane axis was interpreted using a ground-state harmonic oscillator model [14].

## 3. Results and Discussions

After emerging the differential out-of-plane phonon [12] under the influence of the antiferromagnetic spin-density wave, we observed that the average phonon frequency of bulk FeSe is decreased by ~2%. This decrease is accompanied by a relative out-of-plane vibrational amplitude between the two adjacent Fe atoms, which is approximately 0.04 Å. When compared to the norm of $u_{i}(x,y,z)$ in bulk FeSe, typically ~0.15 Å, the differential lattice vibrations $\delta u_{i}(z)$ are significant. The differential orthogonal displacement of lattice ions is observed not only in the FeSe system but also in the FeAs system. For example, the Ba_0.6_K_0.4_Fe_2_As_2_ superconductor [19], where their relative out-of-plane lattice vibrations are ~0.07 Å. These findings suggest that the presence of alternating AFM and SDW ordering triggers the abnormal out-of-plane lattice vibrations in some iron-based superconductors. In particular, the magnitude of $\delta u_{i}(z)$ in FeSe is smaller compared to that in the K-doped BaFe_2_As_2_. This is attributed to the fact that the atomic spring constant of the FeSe bond is approximately two times weaker than that of the FeAs bond, where a weak atomic spring constant is less effective in creating an orthogonal phonon in the tetrahedral zone.

**Figure 1:**
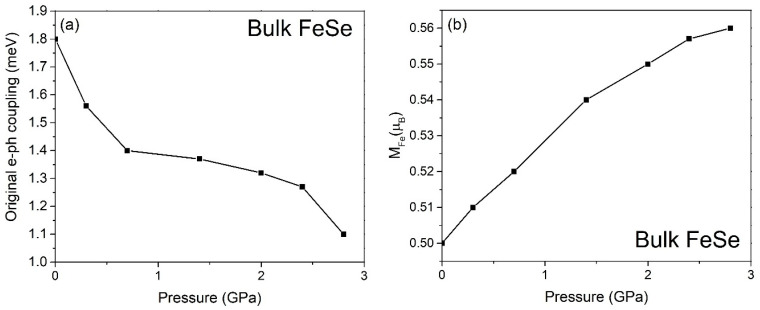
(**a**) The conventional electron–phonon coupling of FeSe. (**b**) The magnetic moment of Fe atoms increases with pressure, where the exchange-correlation energy increases by a few percent upon compression.

**Figure 2:**
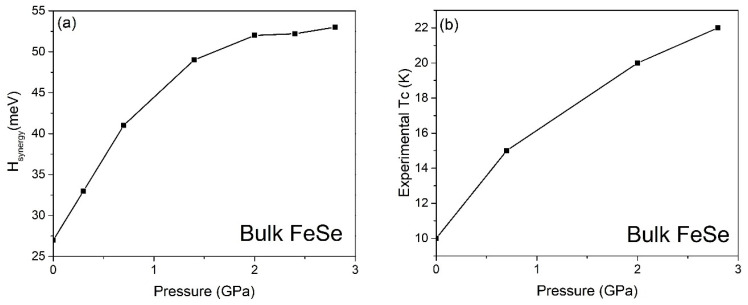
Bulk FeSe; (**a**) antiferromagnetic SDW- and CDW-enhanced electron–differential phonon coupling under the gap anisotropy. (**b**) The T_c_ measured in the experiments [20].

The 11-type iron-based superconductor, bulk FeSe, exhibits a superconducting transition temperature (T_c_) of approximately 10 K under ambient pressure [20]. Notably, the T_c_ of bulk FeSe can be enhanced by applying compression [20]. Our calculations indicate that the bare electron–phonon coupling in bulk FeSe, assuming isotropic momentum space, is determined to be 1.8 meV at 0 GPa. After activating the spin-unrestricted mode in the ab initio calculation, the antiferromagnetically assisted electron–phonon coupling of bulk FeSe raises to 2.7 meV only, where R_AF_~1.2. It is important to highlight that the bare electron–phonon coupling strength exhibits a decreasing trend with increasing pressure, as depicted in Figure 1a. This observation indicates that the antiferromagnetically enhanced electron–phonon coupling alone is insufficient to match both the observed ARPES range and the positive dependence of T_c_ on pressure in Figure 2. However, the exchange factor increases with pressure, which convinces us to combine the spin-density-wave phenomenon with electron–phonon coupling to check if the antiferromagnetically SDW assisted electron–differential phonon coupling stands a chance to match the ARPES energy range. We have found that those synergic effects at the AFM maxima boost the interaction to 10.8 meV. Furthermore, because of Maxwell’s equation, the induced CDW effect strengthens the antiferromagnetically SDW- and CDW-assisted electron–differential phonon coupling to 47.6 meV, where R_CDW_ is justified to be 2.1 in FeSe [12]. As an anisotropic momentum space is observed in FeSe [21], the $H_{synergy}$ under the gap anisotropy is then paled from 47.6 meV to 27.6 meV (step-by-step calculation: please read the Supplementary Materials), which is comparable to the experimental ARPES range [4].

In unconventional superconductors, a reduction in the electron–phonon coupling strength has been observed due to the presence of symmetry in momentum space. Specifically, when a 4-fold symmetry is present in the superconducting gap, the electron–phonon coupling can be diminished by a factor of approximately 0.6–0.8 [22]. Our analysis reveals that the reduction in the $H_{synergy}$ attributed to the gap anisotropy is approximately 0.58 in bulk FeSe, which is consistent with the previous findings reported in the literature. The absence of an isotope effect on the T_c_ in most iron-based superconductors could potentially be attributed to the electron–differential phonon coupling that is triggered by antiferromagnetically ordered spin-density-wave and charge-density-wave phenomena. Even when the iron atom in these compounds carries an additional neutron due to isotope substitution, the differential effective mass of the iron atoms between its nearest neighbors remains unchanged, leading to the insensitivity of T_c_ to isotopic variations.

To validate the comparability between the $H_{synergy}$ and the ARPES energy range, it is essential to select another iron-based compound that exhibits a substantial contrast in the ARPES range. Therefore, our upcoming investigation concentrates on the FeSe/SrTiO_3_ composite with a high T_c_ of ~100 K in which its ARPES range in the experiment covers a broader energy range of ~0.1–0.3 eV [5], allowing for a comprehensive comparison and analysis. Following the same approach, our simulations have revealed that the magnetoelectric pulse is stronger in FeSe/SrTiO_3_ relative to bulk FeSe. This difference arises from the asymmetric structural characteristics of the FeSe/SrTiO_3_ interface. In FeSe/SrTiO_3_, the upper tetrahedral region of FeSe film is exposed to a vacuum environment, while the lower tetrahedral region interacts with the SrTiO_3_ substrate, and between them, there is an interfacial phonon shaping these asymmetric tetrahedral angles. This differential proximity interaction under the interfacial phonon further actuates a more pronounced CDW effect when compared to the bulk FeSe. Also, the spin-density wave is much stronger in FeSe/SrTiO_3_. With these synergetic effects, the antiferromagnetic SDW- and CDW-assisted electron–differential phonon coupling of FeSe/SrTiO_3_ reinforced to ~0.5 eV (step-by-step calculation: please read the Supplementary Materials), falling within the same order of magnitude as the experimental ARPES range. Notably, the experimental observation also demonstrates a significant strength in the electron–phonon coupling in FeSe/SrTiO_3_, with the interfacial phonon reaching as high as 1150 K [23], where its ultra-strong electron–phonon coupling has been reported in this material [5,23,24]. By drawing a parallel between bulk FeSe and FeSe/SrTiO_3_, we observe a proportional relationship between the antiferromagnetic SDW- and CDW-assisted electron–differential phonon coupling and the ARPES energy range.

The coupling between conventional magnetic fluctuations and conventional electron-phonon interactions in Fe-based materials, or in general, may not be directly associated with the unusual electronic states observed in ARPES. However, our findings from studies on iron-based compounds highlight a legitimate concern that the potential synergistic effect on electron–differential phonon coupling may have been overlooked, where the interplay between antiferromagnetism, spin-density-wave, and charge-density-wave phenomena amplifies the interaction between electrons and the periodic negative synergistic potential to a degree that is comparable to the unconventional energy range observed in ARPES data.

Calibrating the numerical parameters to achieve a good agreement with experimental observations can be an important step toward comprehending the underlying phenomena [12,13]. The subsequent step should involve delving into the scientific principles that elucidate why such an agreement is achieved. This deeper exploration aims to uncover the fundamental mechanisms and processes that contribute to the observed phenomenon and provide a robust scientific understanding of the phenomenon in question. We revisit the case: the enormous increase in electron–differential phonon coupling observed in FeSe and FeSe/SrTiO_3_ may be understood through the physics behind the redistribution of spatial antiferromagnetic fluctuations under the effect of spin-density waves. This redistribution leads to the formation of alternating regions of AFM local minima and AFM local maxima between the nearest neighbors of Fe atoms, in which the effect of spin-density waves transfer the antiferromagnetic energy from the AFM local minima to the AFM local maxima per repeating unit. The SDW-induced redistribution of AFM fluctuations maintains the conservation of antiferromagnetic energy, but this energy transfer doubles the local electron–phonon scattering matrix at the AFM local maxima within the repeating unit. The process of AFM redistribution continues over time, periodically switching the locations of AFM local maxima and local minima, resembling an interference-like phenomenon. For instance, at time t, the odd-numbered lattice points represent the AFM maxima, while the even-numbered lattice points correspond to the AFM minima. However, as time progresses to half the SDW period of oscillation, the AFM minima configuration switches to occur in the odd-numbered lattice points, while the AFM maxima configuration appears in the even-numbered lattice points. In other words, the alternating (odd and even) lattice points that form the AFM maxima and minima change their configuration periodically over time. This interference-like effect significantly increases the peak value of the AFM- and SDW-enhanced electron–phonon coupling.

Furthermore, antiferromagnetism always slows down phonons in the lattice [18]. Lattice ions experiencing the AFM local maxima always vibrate slowly, equivalently exhibiting a larger effective atomic mass. On the other hand, the neighboring lattice ions located at the AFM local minima vibrate relatively faster, indicating a smaller effective atomic mass. This differential effective mass among neighboring Fe atoms leads to the variation in their vibrational amplitudes orthogonally [12,15], triggering the differential out-of-plane phonons per repeating unit at the boundary between the non-magnetic and magnetic lattice sites. At this instantaneous time, based on Maxwell’s equation, electrons under a time-varying magnetic field experience an induced electric potential at the boundary, which produces local charges in the form of a charge-density-wave effect. As this process is instantaneous, we refer to it as a magnetoelectric pulse that is further collected by the isotope-insensitive differential phonon (not average phonon) in the system, which interacts with electrons in nature. In reality, AFM is not a mean field in iron-based superconductors, where the AFM energy at the maxima is very strong. Hence, a conventional mean-field DFT approach may not be able to model this magnetoelectric pulse accurately.

The Ising expression [14] is believed to capture the general behavior of how antiferromagnetism changes under pressure, which can make our simulation less dependent on the choice of DFT functional. Although the Ising expression may not provide exact quantitative values, it can still generate a general trend that offers valuable insights into the effects of pressure on antiferromagnetic behavior. Nonetheless, our objective is to observe whether the AFM, SDW, and CDW amplified the electron–differential phonon coupling, as well as the experimental ARPES energy range, exhibit the same order of magnitude. In this context, the accuracy of our work is already sufficient to find out the proportionality between them.

On the other hand, KFe_2_As_2_ is an iron-based superconductor characterized by the absence of magnetic ordering, which does not show an unusual distribution of electrons below the Fermi level experimentally [25]. This lack of magnetic ordering not only suppresses antiferromagnetic fluctuations but also renders the amplification factor R_AF_ ineffective (or R_AF_ = 1). In the absence of antiferromagnetic spin ordering, the spin-density-wave factor R_SDW_ is also irrelevant, leading to R_SDW_ = 1. When SDW order is absent, there is no magnetic-to-non-magnetic boundary for the strong emergence of charge-density-wave (CDW) phenomena. Consequently, the amplification factor R_CDW_ is not applicable here and can be simplified to approximately 1. The exchange factor f(E_ex_) remains at 1, as no external pressure is applied. Ultimately, the *H_synergy_* of KFe_2_As_2_ is approximately 3 meV, which is not strong enough to induce unusual electron distributions in the range of 30 meV to 300 meV below the Fermi level, aligning with the experimental spectrum. In contrast, we have calculated the *H_synergy_* of KFe_2_Se_2_. Given that spin ordering is present [26], R_SDW_ = 2, irrespective of the magnetic ordering pattern at neighboring sites. Our calculations indicate that the charge-density-wave (CDW) effect exists, with R_CDW_ calculated as 1.03 and the antiferromagnetic amplification factor (R_AF_) at ~1.2. Consequently, the *H_synergy_* of KFe_2_Se_2_ is ~14 meV. The exchange factor f(E_ex_) remains at 1, as no external pressure is applied. In the experimental spectrum, K_0.73_Fe_1.6_Se_2_ also exhibits a slightly unusual electron distribution below the Fermi level [6], although this is not as pronounced as FeSe/SrTiO_3_. In this context, we note that FeSe, KFe_2_Se_2_, and FeSe/SrTiO_3_ exhibit unusual electron distributions below the Fermi level, while KFe_2_As_2_ does not. This observation may suggest that the presence of an antiferromagnetic spin-density wave, in conjunction with a charge-density wave on electron–differential phonon coupling, may facilitate the emergence of unusual electron distributions.

While we may have identified one of the factors influencing ARPES energy, accurately calculating the ARPES energy range remains an open question due to numerous hidden parameters that require further investigation. In the case of FeSe, the gap anisotropy reduces the *H_synergy_* by approximately 40%, suggesting that the ARPES range may be linked to the symmetry of the superconducting gap (e.g., s, d, or chiral). On the other hand, heterostructures are likely to play a role in tuning the ARPES range. For example, the FeSe film on SrTiO_3_ shows ~6-fold increase in the AFM fluctuations due to surface strain. Furthermore, the R_CDW_ value increases from 2.1 to 2.9 [27,28]. All these parameters, together with, but not limited to, the strength of spin–orbit coupling and nematicity, could contribute to a more accurate calculation of the ARPES energy range despite the fact that the analytic solution for this problem is still unsolved.

In our investigation of the impact of spin–orbital coupling on the $H_{synergy}$ [29], we observed that regardless of whether we include the spin–orbital coupling in our calculations or not, the changes observed in the $H_{synergy}$ of these samples are only around 10%. However, it is unfair if we underestimate the effect of spin–orbital coupling on the pairing mechanism of iron-based superconductors. The reason is that, although the $H_{synergy}$ and the ARPES energy range may be correlated, it is still an open question whether spin–orbital coupling is one of the ingredients that pulls a trigger on activating iron-based superconductivity [29]. Nematicity and spin–orbit coupling are thought to give rise to an anisotropic Fermi surface [29], which may also influence the specific energy range below the Fermi level.

AI research on iron-based superconductivity has become a hot topic. This involves examining the contrasts among different iron-based superconductors to identify the correlations between various variables and output. In our work, we have discovered a proportional relationship between synergistic energy and the ARPES energy range. Indeed, we acknowledge that many factors contribute to the ARPES energy range (not just AFM, SDW, and CDW). This proportional relationship should be considered in the training data when using AI [9,10,11] to weigh the parameters associated with the ARPES energy range.

## 4. Conclusions

Our research demonstrates that the isotope-insensitive electron–differential phonon coupling, amplified by antiferromagnetism, spin-density waves, and charge-density waves, closely matches the experimental magnitudes recorded in the ARPES energy range. In addition, we observed a significant correlation between this coupling and the ARPES energy range in both the FeSe and FeSe/SrTiO_3_ samples. These results imply that the magnetoelectric pulse linked to spin-density waves and charge-density waves may play a role in triggering the ARPES energy range.

## Data Availability

The data presented in this study are available on request from the corresponding author. The data are not publicly available due to privacy.

## Supplementary Materials

The following supporting information can be downloaded at: https://www.mdpi.com/article/10.3390/ma17215204/s1, Step-by-step calculation of the synergistic energy.
