# Supplementary material for: Exploring Unconventional Electron Distribution Patterns: Contrasts Between FeSe and FeSe/STO Using an Ab Initio Approach
Source: Materials (Basel). 2024 Oct 25;17(21):5204. doi: 10.3390/ma17215204 (PMC11547900; doi:10.3390/ma17215204)
Supplement: Supplementary file 1 [file materials-17-05204-s001.zip › materials-3253919-supplementary.pdf]

Supplementary materials

These are the key steps for calculating the amplification factors for $H_{synergy.}$

1) Obtain R_{AF}^2 by taking the ratio of the dimensionless electron–phonon coupling in spin-unrestricted (magnetic) and spin-restricted modes (non-magnetic).

2) The momentum-dependence of k-space is simulated under spin-unrestricted mode (i.e., the task of Fermi-surface simulation)

3) Through comparing the magnetic moment and exchange energy at different pressures P, the exchange factor $f(E_{ex})$ is obtainable for $P > 0$

4) $R_{SDW} = 2$ is obtained from the analytical results validated by ref 16. DFT software is not needed.

5) The calculation of R_{CDW} can be treated by estimating the effective electronic density of states. The details are as follows:

To compute R_{CDW}, any DFT software with the ability to calculate the electronic density of states is fine. We applied the two-channel model [27,28] that superimposes the effects of the upper and lower 'channels' in the unit cell separately.

Here, the 'upper channel' means that the tetrahedral atom in the lower tetrahedral region is removed in the unit cell [27,28]. Similarly, the 'lower channel' means that the tetrahedral atom in the upper tetrahedral region is removed in the unit cell [27,28].

Then, calculate the electronic density of states DOS at the Fermi level for the upper and lower channels separately, and then take the average of these two values, which emerge from the CDW effect of the abnormal out-of-plane phonon before it is masked by the mean-field superposition of out-of-plane phonon [12,27,28]. This average $\langle DOS(E_F)\rangle$ per atom is then divided by the electronic density of the states at the Fermi level $DOS(E_F)$ per atom in the conventional repeated unit that includes both the upper and lower tetrahedral atoms per layer [27,28], in which $\langle DOS(E_F)\rangle / DOS(E_F)$ becomes R_{CDW}.

This two-channel model effectively simulates the charge-density wave without the need for time-consuming experimental calibration [12], yielding results that closely match those of the FeSe system (for example, $R_{CDW} = 2.2$ from experiments [12] and $R_{CDW} = 2.1$ from the model [27,28]).

Since R_{AF} and R_{SDW} have taken antiferromagnetic ordering into account, the R_{CDW} and the electron–phonon coupling should be estimated under spin-restricted mode.

Example calculations:

- Bulk FeSe

Step 1: Assume the k-space is isotropic. Calculate the isotropic non-magnetic electron–phonon coupling by the Jellium model (i.e., 1.8meV for bulk FeSe, where the electron–phonon coupling of most ordinary materials is in the order of meV).

Step 2: Multiply the isotropic non-magnetic electron–phonon coupling by $R_{AF}^2 = 1.24^2$ together with the replacement of the anisotropic k-space in the integral (i.e., 1.04meV x 1.24^2 = 1.6meV, where the anisotropic k-space reduces the electron–phonon coupling by the ratio of 1.04/1.8=0.58). This is called AFM-assisted anisotropic electron–phonon coupling.

Step 3: Multiply the AFM-assisted anisotropic electron–phonon coupling (1.6meV) by the analytic solution of $R_{SDW}^2 = 2^2$ (i.e., 1.6meV x 2^2 = 6.4meV) [12]. This is called the AFM–SDW-assisted anisotropic electron–phonon coupling.

Step 4: Multiply the AFM–SDW-assisted anisotropic electron–phonon coupling (6.4meV) by the experimentally calibrated CDW parameter of $R_{CDW}^2 = 2.2^2$ (i.e., 6.4meV x 2.2^2 = 30meV) [12]. This is called the AFM–SDW–CDW-assisted anisotropic electron–differential phonon coupling.

After this, the $H_{synergy}$=30meV is obtained.

- FeSe/$SrTiO_3$

Step 1: Assume the k-space is isotropic. Calculate the isotropic non-magnetic electron–phonon coupling by the Jellium model (i.e., 0.8meV).

Step 2: Multiple the isotropic non-magnetic electron–phonon coupling by $R_{AP}^2 |_{P>0} = R_{AP}^2 |_{P=0} \cdot f(E_{ex}) = 1.23^2 \cdot (\frac{1.2^2}{0.6^2} 1.14) = 6.8$, together with the replacement of anisotropic k-space (i.e., 0.45meV x 6.8 = 2.6meV) in the integral, where the anisotropic k-space reduces the electron–phonon coupling by the ratio of 0.45/0.8=0.56. This is called AFM-assisted anisotropic electron–phonon coupling.

Step 3: Multiply the AFM-assisted anisotropic electron–phonon coupling (2.6meV) by the analytic solution of $R_{SDW}^2 = 2^2$ (i.e. 2.6meV x 2^2 = 10.4meV) [12]. This is called the AFM–SDW-assisted anisotropic electron–phonon coupling.

Step 4: Multiply the AFM–SDW-assisted anisotropic electron–phonon coupling (10.4meV) by the CDW parameter of $R_{CDW}^2 |_{P>0} \sim R_{CDW}^2 ||_{P=0} \cdot f(E_{ex}) = 2.9^2 \cdot (\frac{1.2^2}{0.6^2} 1.14) = 38.3$. Then, it becomes 10.4meV x 38.3 = 455meV) [12]. This is called AFM–SDW–CDW-assisted anisotropic electron–phonon coupling.

After this, $H_{synergy}$=455meV is obtained.
